# *Shinmi* (親身): a Distinctive Japanese Medical Virtue?

**DOI:** 10.1007/s41649-023-00261-6

**Published:** 2023-11-04

**Authors:** Reina Ozeki-Hayashi, Dominic J. C. Wilkinson

**Affiliations:** 1 Oxford Uehiro Centre for Practical Ethics, Faculty of Philosophy, University of Oxford, Oxford, UK; 2 University of Tokyo, Tokyo, Japan; 3 John Radcliffe Hospital, Oxford, UK; 4 Murdoch Children’s Research Institute, Melbourne, Australia; 5 Centre for Biomedical Ethics, Yong Loo Lin School of Medicine, National University of Singapore, Singapore

**Keywords:** Patient-doctor relationship, Virtue ethics, Emotional involvement, Cultural diversity, Professionalism

## Abstract

In Western countries, the ideal professional and ethical attributes of healthcare providers and the ideal patient-doctor relationship have been analysed in detail. Other cultures, however, may have different norms, arising in response to diverse healthcare needs, cultural values and offering alternative perspectives. In this paper, drawing a case study, we introduce the concept of *Shinmi*, used in Japan to describe a desirable approach to medical care. *Shinmi* means kind or cordial in Japanese. In the medical context, it refers to doctors treating patients with a degree of emotional closeness as if they were the doctors’ own family. We analyse the concept of *Shinmi*, drawing on virtue ethics. We distinguish two different elements to a *Shinmi-na* attitude. As illustrated in our example, excessive *Shinmi* can be problematic for patients and doctors. Furthermore, elements of *Shinmi* may conflict with existing Western values (for example, norms that encourage emotional detachment and discourage doctors’ treatment of family members). However, if pursued appropriately, we argue that a balanced *Shinmi*-na approach can be conducive to the goals of medicine. The concept of *Shinmi* may be valuable for medical students, in Japanese and potentially other health care systems, and help them to cultivate a virtuous approach to meeting the emotional needs of patients.

## Introduction

Much has been written about the ideal doctor-patient relationship and doctor’s attitudes in terms of professionalism and ethics, mainly from the West. Although there has been less analysis of the concept of a “good doctor” in other cultures, different contexts, social values and healthcare systems might give rise to different norms. The preferences of both doctors and patients will also potentially vary depending on the cultural background (Sheeran et al. 2023; Schouten and Meeuwesen 2006). In many countries’ medical education systems, concepts from Western countries are imported and translated. However, it is possible that distinctive national professional norms might arise that complement or compete with those Western ideals.

In Japan, one term used by professionals and the public to describe the ideal form of medical care is *Shinmi* (親身). The dictionary definition of this word includes several distinct meanings. First, it refers to a person related by blood or a blood relationship. It also refers to taking somebody to one’s heart, e.g. look after somebody with tender care. The related adjective *Shinmi-na* means (1) amiable, cordial, (2) kind, good-hearted and (3) benevolent, open-hearted, etc.

Kindness or benevolence appear similar to the desirable attitudes of healthcare providers in Western countries (Pellegrino 2002). However, as the other definitions, such as “tender care like a family”, suggest, *Shinmi* is not merely kindness but also implies a level of care for patients as if they were doctors’ own family members. It suggests a psychological proximity and a potential blurring of professional boundaries.

In this paper, we will introduce the concept of *Shinmi* in Japanese healthcare through a fictionalised case that includes a doctor’s *Shinmi-na* attitude. We will define the key elements of *Shinmi* in healthcare and identify some potential problems. Finally, we will analyse *Shinmi* through a virtue ethics lens and make suggestions for how to avoid excessive *Shinmi* and maintain a balance between the needs of the patient, other patients and the doctor in medical practice.

## Case^1^

When Doctor B was a young doctor, she was responsible for a female patient, Y, in her 50s with gastric cancer and peritoneal metastases. The patient had been admitted to a hospice as further curative treatment was deemed to be futile. Y had a husband and a daughter at university. She was Dr B’s first patient on hospice duty, and Dr B was nervous but hopeful that she could manage Y’s distress. During Y’s stay in the hospice, her pain gradually worsened; she was slowly dying. Y wanted to spend time with her family, but her daughter and husband could not accept that she was dying and rarely came to see her.

Dr B had a good relationship with Y. She always described Dr B as a “*Shinmi-na* doctor”. During her residency, her supervisor had taught her to “treat patients as if they were your own family”, and Dr B was pleased that Y trusted her, and that she had established what she saw as the ideal relationship with her patient. Dr B spent more time at Y’s bedside than with any other patient, and they began to talk a lot about things other than work. As Y’s family distanced themselves, B found herself becoming angry and resentful towards them, and felt that she had to support Y instead of Y’s family, who seemed cold-hearted. When Y had to make a difficult decision, she asked B: “If it was your mother’s case, what option would you choose?” When nurses could not find Dr B on the ward, they would sometimes go to Y’s room imagining that Dr B would be there.

Y was fully aware of her medical condition and had already indicated her intention not to receive cardiopulmonary resuscitation (CPR). Dr B wondered if it might be better to prolong Y’s life with CPR, even against her wishes, to give Y a chance to spend as much time with her family as possible (Dr B resisted this temptation). Ultimately, Y died without her family having enough time to spend with her. After Y’s death, Dr B felt a terrible loss and was mildly burn-out. Dr B left the palliative care unit 2 years later.

In caring for Y, Dr B provided *family-like*, dedicated and special care for Y, which she did not provide to other patients. Dr B reacted strongly to and shared her patient’s emotions and developed strong negative feelings towards Y’s family. In retrospect, Dr B acknowledged that at the time, she did not realise the importance of psychological distance from her patient and did not achieve a proper balance between emotional involvement and clinical detachment. She lost her professional objectivity before the patient’s death and this contributed to her becoming burnt out. Dr B had tried to be the *Shinmi-na* doctor, the ideal in Japan, but why did it end up this way?”.

We will start by considering what constitutes a *Shinmi-na* doctor.

## *Shinmi-na* (親身な) Medicine/Doctor

In Japan, medical professionals with a *Shinmi-na* attitude are considered virtuous practitioners. In addition, many Japanese patients understand *Shinmi-na* medical care as ideal. For example, a search of hospital and clinic websites identified a number of institutions who explicitly described themselves using this adjective (Table 1).

**Table 1:** *Shinmi* in the description of medical institutions’ policies

1	We aim to provide *Shinmi-na* medical service and cordial medical care	H Clinic
2	We provide high-quality, *Shinmi-na* medical care, considering our patients like our family	O Public Hospital
3	In all our practices, we do our best to communicate with our patients and treat them with *Shinmi-na* care like a family	A clinic
4	We aim to provide *Shinmi-na* medical care for everyone. We always provide *Shinmi-na* medical service for every patient by responding to patients’ physical and psychological concerns like a family	O Clinic

Google search “親身な医療 *Shinmi-na* medicine AND 親身な病院 *Shinmi-na* Hospital” (Accessed 21 May 2023)

A separate search of patient comments and complaints left online yielded a number of characteristics of either *Shinmi* and non-*Shinmi* doctors (Table 2).

**Table 2:** Attitudes relating *Shinmi* in patients’ comments and complaints online

*Shinmi-na* doctor	Non-*Shinmi-na* doctor
- Sees the patient as a whole person	- Only looks at the computer screen and does not look at the patient
- Heals the mental condition as well as the physical condition	- Only talks about the disease
- Listens carefully	- Only 3 minutes to see the patient at the outpatient clinic
- Dr went to another hospital himself and gave information about his patient to the new attending doctor during non-working hours	- Dr did not listen to me
- Dr contacted the hospital about me even on her day off	
- Treats me like his own family	
- Dr cried and performed CPR on my mother	

Google search “{親身(*Shinmi*) OR 親身ではない(Non-*Shinmi*)} AND 医師(Doctor) -患者の声 or コメント (patient comments or voice)” (Accessed 21 May 2023)

These results support the idea that Japanese patients perceive a doctor’s *Shinmi-na* attitude as “the doctors’ familial and emotional involvement as if they were patient family”. The term is used to describe hospital policy or values and is also used by patients as a mark of a good doctor.

However, as the case example illustrates, if a doctor tries to be an overly *Shinmi-na* doctor, it can be detrimental to both themselves and to their patients. In the next section, we will describe two separate elements of a *Shinmi-na* doctor and identify the problems that can arise if this is pursued excessively.

## *Shinmi-na* Attitude

### 1. “Familism”

One clear element of the meaning of *Shinmi* is that the doctor treats the patient as if they were a member of the doctor’s family. We could refer to this as “Familism”—a term that indicates a cultural value placed on close family relationships and prioritisation of family members (Kuzuu 2007). In Japan, this may reflect the influence of Confucian and neo-Confucian values, which arrived from China in the Asuka period (fifth and sixth centuries), but were embraced and disseminated especially in the seventeenth and eighteenth centuries (Chou 2002). In Confucian society, the community of significance is the family and its extension, the kinship community. In other words, the stronger the blood-related ties, the stronger the degree of care (Kuzuu 2007).

One form of familism is when health professionals treat their own family members. The permissibility of this varies from country to country. There are no guidelines or laws against doctors treating family members in Germany (Mücke et al. 2022), Malaysia (Nik-Sherina and Ng 2006), Japan (Matsunaga et al. 2022) and other Asian countries (Ooi 2018). In Japan, Matsunaga et al. reported that about 80% of family physicians had treated their own family members, and almost 90% were satisfied with their behaviours (Matsunaga et al. 2022).

On the other hand, in many Western countries, guidelines prohibit or strongly recommend against doctors treating their own family members (American Medical Association Council 2012; General Medical Council 2013). Reasons for this prohibition include the risk of (1) losing objectivity and blurring of professional boundaries between patients and doctors, (2) compromised continuity of care, (3) doctors making errors in medical judgement, (4) necessary information not being communicated due to preconceptions of the patients or reluctance to deal with sensitive content, (5) overmedication or under-medication and (6) problems with confidentiality (Fromme et al. 2008; Ooi 2018).

One instantiation of familism that may be familiar to Western doctors is when a patient asks the doctor, “If the patient were your family member, which treatment would you choose?” (Truog 1999) This question can be gratifying for doctors because it shows that patients trust their doctors. For patients and their families, asking this question to the doctor is also meant as a request to certify the doctor as the authority in the family member’s care, to have the doctor take part of the moral burden of making difficult decisions, and to be more involved in the patient’s care. However, Truog states that a doctor meeting such demands is risky: “Physicians are experts in medicine, but not in patient’s values. Doctors should address this question carefully, as it relates to the more general and controversial area of defining the boundaries between doctor and patient”. Truog warns about the potential for doctors to lose their objectivity.

Further criticisms of “Familism” include an inherent tension (and potential contradiction) in the concept of treating patients in a way that is similar to the treatment of family members. Within familism and within a Confucian norm of family relationships, is the idea of *special* treatment of family members—giving them attention and care over and above the care provided to others (Kuzuu 2007). In this approach, familism takes precedence over the interests of society as a whole, leading to a lack of attention to the public good. However, this might contradict other ethical duties—for example the need to distribute limited healthcare resources equitably (as well as to achieve the greatest good). Excessive *Shinmi* raises problems from the perspective of equity. Furthermore, this approach to providing *special Shinmi* patient care may ultimately be self-defeating. Treating every patient in the way that family members are treated would mean that family members (and non-family patients) no longer receive *special* care.

### 2. “Emotional closeness”

Another element of the meaning of *Shinmi* is the doctor’s more emotional involvement with the patients. If *Shinmi-na* doctors see their patients as family members, this can potentially trigger the same emotional reactions to the patient’s situation that they feel towards their family members. For example, in ordinary circumstances, doctors would not necessarily be unduly affected by learning that their patient has been diagnosed with cancer. However, if they learn that their wife or parent has been similarly diagnosed, it is natural that they would be strongly affected psychologically. If the doctor has a *Shinmi-na* relationship, the same responses may be evoked.

Emotional engagement with patients in clinical practice has been discussed in the context of trust and communication with the patient. Medical professionals are often expected to communicate calmly, keep their emotions at bay and reduce their emotional reactions (Coulehan and Williams 2003). In particular, there have been numerous studies on empathy, positioned as necessary in healthcare for building trust with patients and facilitating healthcare. Empathy is sometimes divided into affective empathy, the subjective experience or sharing of another person’s feelings, and cognitive empathy: “the ability to identify and understand the feelings and perspectives of others from an objective standpoint” (Jeffrey 2016). Affective empathy is said to be more likely to be felt towards those in close personal relationships, and those similar to you. *Shinmi-na* doctors feel they have a psychologically closer relationship with their patient, akin to family members and this may trigger affective empathy.

However, Jodi Halpern argues that affective empathy should be avoided in medicine. She contends that when doctors use affective empathy, their emotional reactions reduce the quality of cognitive understanding of patients, impede objectivity, weaken the ability to provide effective care in difficult situations and increase the risk of physician burnout (Halpern 2011). In particular, working with patients with affective empathy over a long period may prevent doctors from seeing the situation from the professional distance necessary to identify the best option for the patient, and they may try to provide all possible treatment (Malbois and Hurst-Majno 2023).

This characteristic of affective empathy also means that empathy is not provided according to the need of the patients (Slovic 2007). There are criticisms that affective empathy is partial and biased and is not offered fairly to all patients (Batson et al. 1995; Bloom 2016).

## A Virtue-Ethics Approach to *Shinmi*

How should we assess *Shinmi* ethically? *Shinmi* is not an ethical principle, rule or duty. Rather, it appears to be a cluster of attitudes and behaviours. It is a potential characteristic of ethical doctors. Moreover, as already noted in the case and in the comments of patients, it may be over or under-expressed. In that sense, analysing *Shinmi* through the lens of virtue ethics may be helpful.

Virtue ethics is often distinguished in its emphasis on virtues, or moral character, in contrast to approaches that emphasises duties or rules (deontology) or the consequences of actions (consequentialism) (Hursthouse and Pettigrove 2022). Edmond Pellegrino (2002) has argued that the traditional professional medical norms were virtue-based, linking good physicians to specific character traits. Citing Aristotle’s moral virtues (which on Aristotle’s account were to be pursued neither excessively, nor insufficiently), he defined an excellent (virtuous) physician as one who most effectively achieves the goals of medicine and exhibits traits essential to achieving those goals. Pellegrino’s influential account included six specific virtues of doctors: fidelity to trust, honesty, compassion, effacement of self-interest, courage and justice. No indication of familism, an element of *Shinmi*, is listed here, but emotional involvement is mentioned in the form of compassion, which refers to the need to enter the patient’s predicament and feel their suffering (akin to cognitive empathy).

While Pellegrino’s virtues are grounded in Western culture, there are also physician virtues that have been described in Eastern culture. Some of these include similar elements to *Shinmi*. According to Daniel Tsai and colleagues, ancient Chinese medical ethics were derived from Confucian virtue ethics. The central theme of this is “仁 (Ren)”, with family values and filial piety, particularly being the root of benevolence (Tsai 2005). As evidence of the influence of this, it has been suggested that the book “Bushido”, written by Inazo Nitobe (2012), which described the way of the Samurai and has its origins in Buddhism, Confucianism and Shintoism, has influenced professionalism in Japanese medicine. Bushido is similar to chivalry or noblesse oblige in the West. In Bushido, seven virtues are advocated. Rectitude (義 Gi), Courage (勇Yu), Benevolence (仁Jin), Politeness (礼 Rei), Honesty (誠Rei), Honour (名誉 Meiyo) and Loyalty (忠義 Chugi) (Nishigori et al. 2014). Of these, “仁 (Jin)” and “忠義 (Chugi)” are similar to concepts of *Shinmi*. “仁 (Jin) Benevolence”, which means love and compassion towards others, is an attitude of emotional involvement and empathy towards others. According to a survey of Japanese physicians, many stated that “仁 (Jin)” is an integral part of medicine. Furthermore, “忠義 (Chugi) Loyalty” prioritises loyalty to the needs and interests of the group, such as a family, in contrast to Western individualism.

In analysing *Shinmi* through the lens of virtue ethics, we could find similarities between the elements of physician virtue and *Shinmi*. On the other hand, in the previous section, we described that elements of *Shinmi* potentially contrast or conflict with the traditional medical ideals in the West. However, the importance of familism and emotional involvement has recently been recognised or reassessed in Western cultures.

The first element, familism, appears particularly desirable in cultures with a Confucian regard for the importance of family. However, many patients, even outside Japan, regard being treated like family by healthcare professionals as something that medical care should aspire to. Perhaps people believe that a doctor’s family member receives better care than a doctor’s non-family members (Abbate 2014)? In the UK, the “Friends and Family Test” is used to assess healthcare quality, asking people (including those who work in the National Health Service) whether they would be willing to recommend a healthcare provider to a family member or friend if they were in the same situation. It suggests that the level of healthcare that would be sufficient to refer to one’s loved ones may be considered the minimum required (NHS England Insight Team 2014).

The second element, affective closeness has also been re-evaluated. Although, as noted, analysis of doctors’ empathy has previously suggested that doctors should avoid affective empathy (Halpern 2011; Hojat et al. 2009), Michalec et al. express concern that focusing too much on cognitive empathy to the exclusion of emotional empathy ultimately might have a negative impact on patient care (Michalec and Hafferty 2022). Such an approach may lead to overemphasis on clinical knowledge, clinical detachment and scientific rationality. Wong et al., in a study of exploration of oncologists’ professional emotional experiences, found that some professionals reported engaging more emotionally with their patients to meet their affective needs and perceive it as an essential strength of being a doctor (Wong et al. 2020). In this research, doctors attempted to strike an appropriate balance between keeping themselves as objective and competent doctors and engaging more emotionally with their patients.

From our analysis of *Shinmi* through the virtue ethics lens, we argue that *Shinmi* can potentially include desirable character traits if doctors properly pursue *Shinmi*. What should doctors look for to achieve the golden mean of *Shinmi* in healthcare?

The risks common to the two elements of *Shinmi* are loss of professional objectivity due to the blurring of professional boundaries with the patient and lack of equity as a public servant. Additionally, affective closeness can undermine the mental well-being of the doctor.

Firstly, to keep professional objectivity, as it has been suggested that self-reflection is necessary in terms of an awareness of the boundaries between doctor and patient for ethically sound clinical practice to take place (Kelly et al. 2003; Malbois and Hurst-Majno 2023). Wong and Fromme suggested two reflective questions, “Am I being a professional?” and “Am I acting professionally?” are essential questions to ask doctors, themselves when performing emotional labour (Fromme et al. 2008; Wong et al. 2020). These questions are also important to maintain professional justice and proper emotional distance with the patient.

Secondly, for balanced emotional involvement, sharing emotional experiences with colleagues, friends and family may support doctors to reflect on their feelings (Kerasidou et al. 2021). Kerasidou et al. also suggested that it is not enough to support the individual for the doctor to pursue the virtue appropriately, but it is also essential to create a supportive work environment that does not hinder the expression of the virtue. They mentioned that doctors could learn and acquire virtuous characters, but virtuous development is hindered if there is no place or opportunity to practise virtue.

## Conclusions

In this paper, we have introduced and analysed a concept, *Shinmi*, that is used by both the public and professionals to capture an ideal of medical practice in Japan. Through our case example, and analysis, we have drawn out two distinctive elements of *Shinmi*: “Familism” and “Emotional closeness”. These elements are somewhat in contrast to and in perhaps conflict with Western medical ideals of impartiality and objectivity. Nevertheless, we have argued that if adopted to the right degree *Shinmi* does appear to capture important and desirable character traits that are conducive to the goals of medicine.

Elaborating and clarifying the concept of a virtue of *Shinmi* may be useful for medical education. Pellegrino refers to Aristotle, “We learn by practice and that the best practice is to follow a model of the virtuous person. In medicine, this means we need virtuous physicians as teachers” (Pellegrino 2002). Medical students in Japan should be taught, as one of us was, to treat their patients as if they were their own family. Learning *Shinmi* may be particularly important to meeting the special emotional needs of patients influenced by Confucian values, who seek out health professionals in a familial relationship. However, professionals also need to appreciate the importance of balancing this against objectivity (and the patient’s own well-being), equity (and the well-being of other patients) and prudence (taking care of their own physical and mental health).

We have drawn on our personal and professional experience, but future empirical work would be valuable to explore how *Shinmi* is currently conceptualised and implemented in Japan. It would be interesting and valuable to investigate what specific attitudes make patients (in Japan or elsewhere) feel *cared for like family*. It would be illuminating to see whether there are parallel virtues in other countries influenced by Confucianism. We have suggested that this concept may have wider relevance. However, there is a need for further analysis to explore whether and how the concept of *Shinmi* could contribute to improving the patient-physician relationship in the West.

## Data Availability

Not applicable.

## Footnotes

1 This is a fictionalised composite case drawing on the experience of the authors.
